# Factors Affecting Learning Satisfaction in Face-to-Face and Non-Face-to-Face Flipped Learning among Nursing Students

**DOI:** 10.3390/ijerph18168641

**Published:** 2021-08-16

**Authors:** Mi-Kyoung Cho, Mi Young Kim

**Affiliations:** 1 Department of Nursing Science, Chungbuk National University, 1 Chungdae-ro, Seowon-gu, Cheongju 28644, Korea; ciamkcho@gmail.com; 2 College of Nursing, Hanyang University, 222 Wangsimni-ro, Seongdong-gu, Seoul 04763, Korea

**Keywords:** learning satisfaction, flipped learning, self-directed learning readiness, professor–student interaction, nonverbal communication, education, distance, exploratory behavior, biometry, Republic of Korea, problem-based learning

## Abstract

Factors influencing students’ learning satisfaction may differ between face-to-face and non-face-to-face flipped learning. For non-face-to-face flipped learning, which was widely employed during the COVID-19 pandemic, it is necessary to examine the impacts on learning satisfaction, which may vary depending on professor–student interaction rather than individual competencies, such as SDL readiness. This descriptive study, conducted 2 March 2019 to 24 June 2020, included 89 s-year, flipped-learning nursing students (28 face-to-face, 61 non-face-to-face). Students completed questionnaires about learning satisfaction, SDL readiness, and professor–student interaction. The data, collected using e-surveys, were analyzed using descriptive statistics, *t*-test, ANOVA, Pearson’s correlation, and multiple stepwise regression with IBM’s SPSS Statistics 25.0 program. The total average score of learning satisfaction (38.19 ± 6.04) was positively correlated with SDL readiness (*r* = 0.56, *p* < 0.001) and professor–student interaction (*r* = 0.36, *p* = 0.001), although total learning satisfaction was significantly different between the face-to-face and the non-face-to-face groups (*t* = 5.28, *p* = 0.024). They were also significant influencing factors, along with face-to-face flipped learning, for total learning satisfaction (*F* = 18.00, *p* < 0.001, explanatory power = 36.7%), suggesting flipped learners in non-face-to-face contexts must increase engagement beyond professor–student interaction.

## 1. Introduction

### 1.1. Study Rationale

In the era of the COVID-19 pandemic, the role of nurses with hands-on experience has become important, and it has become critical for nursing education institutions to train nurses with the competency to perform duties in an actual clinical setting. Accordingly, there is a call for the transformation of university education, including the education of nurses who can demonstrate competency in complex clinical settings [1]. In a situation where improving the quality of university-level education is required, face-to-face classes were impossible due to the COVID-19 pandemic, and non-face-to-face classes became inevitable [2]. As classes transitioned to online models, lack of university resources or confusion, such as system server instability and learning-management system failure, became problematic issues, and improvement in this regard has become necessary [3]. Such a sudden shift in circumstances revealed concerns about aspects of the educational system, such as deterioration in the quality of university-level education [4] However, in addition to the systematic problems that occurred in the process of transitioning to a non-face-to-face class format in recent years, the sudden complete transition to non-face-to-face classes brought difficulty and confusion, which was a sudden change in learning method without a preparation period, due to unpredictable situations for students.

Learners who experienced a sudden transition to online classes due to the COVID-19 pandemic experienced low levels of class satisfaction and class efficacy [5] as well as difficulties, such as using efficient time management, decreased concentration, maladaptation to online learning, and the absence of communication between the learner and the instructors [6]. Further, the fact that there is no physical place for the class and that the time could be adjusted flexibly meant that the students’ autonomy increased, but self-directed learning in this environment became even more important.

The importance of self-directed learning ability has been emphasized even before the COVID-19 pandemic. Universities introduced and applied flipped learning [7], emphasizing learner-led learning instead of unilateral teaching and learning. Flipped learning is a teaching and learning method in which students listen to lectures individually using online and digital contents outside the classroom and perform various learning activities, including assignments, in the classroom. It is a method of participating in learner-centered, interactive lessons, such as problem-solving activities or discussions in the classroom [8]. As effects of flipped learning, improvements have been reported for academic achievement and satisfaction [9,10], class participation and interest [11], and self-efficacy [12].

Currently, all university-level education is being conducted non-face-to-face due to the COVID-19 pandemic. However, even after the COVID-19 pandemic is over, non-face-to-face classes may still need to be conducted due to similar circumstances. Therefore, various face-to-face teaching and learning methods should also be available to be implemented non-face-to-face. A typical form of flipped learning is a combination of non-face-to-face pre-learning and face-to-face learning activities. Therefore, it is necessary to examine the effects of implementing flipped learning completely non-face-to-face, that is, when the in-class course has been flexibly transformed as non-face-to-face and when various types of flipped learning are implemented. One essential aspect of transitioning to a new method of teaching is learning satisfaction, which is a state of mind obtained when the learner has achieved the purpose of learning or the individual learner’s expectations are met [13]; it has factors that could potentially affect learning performance. Owing to the fact that learning satisfaction with the class of college students is based on the evaluation of the quality of college education and improvement of academic ability [14], it can be seen that learning satisfaction has a comprehensive meaning, including achievement. Therefore, it is necessary to check the level of satisfaction when a new learning method is introduced and identify the factors that affect this satisfaction. 

One such factor is the self-directed learning readiness emphasized in flipped learning. Self-directed learning readiness is a key factor in flipped learning; thus, it is necessary to identify whether there is a difference in self-directed learning readiness when the in-class content is delivered face-to-face or non-face-to-face and whether this could affect learning satisfaction. Another factor that could affect satisfaction is the interaction between the instructor and the learner, which is emphasized in flipped learning [7]. However, what each learner actually feels and their perceived level of intensity of the interaction may vary [15]. The most representative characteristic of online education is that, unlike face-to-face educational activities, all interactions must rely on the medium used [16]. Furthermore, it is necessary to analyze the differences in interactions between face-to-face and non-face-to-face learning environments and online interactions [17]. 

Arguably, advancements in IT technology demand the shift toward dismantling schools’ boundaries, removing division between subjects, doing away with traditional educational culture, changing the role of instructors and learners, and moving toward space-oriented rather than location-oriented education [18], and the pandemic has accelerated these changes. Although the COVID-19 pandemic may be over someday, similar scenarios may require non-face-to-face implementation. Flipped learning is flexible, as its form can be changed, and classes can be operated in various ways. In this study, we sought to examine the effects of face-to-face flipped and non-face-to-face flipped learning satisfaction, self-directed learning readiness, and professor–student interaction and identify the factors that affect learner satisfaction when flipped learning is used as a teaching method. In addition, we intended to investigate the flexible adaptability of flipped learning to provide foundational data for future use in configuring various teaching methods. 

### 1.2. Objectives

This study was conducted with nursing students to examine the effects of face-to-face flipped learning and non-face-to-face flipped learning on learning satisfaction, self-directed learning readiness, and professor–student interaction. We also sought to identify the factors that affect learner satisfaction. Specific objectives are as follows:

1. Identify the differences in learner satisfaction, self-directed learning readiness, and professor–student interaction between face-to-face flipped learning and non-face-to-face flipped learning;
2. Identify learner satisfaction when flipped learning is used as a teaching and learning method;
3. Identify the correlation among learner satisfaction, self-directed learning readiness, and professor–student interaction when flipped learning is used as a teaching and learning method; and
4. Identify the factors that affect learner satisfaction when flipped learning is used as a teaching and learning method.

## 2. Materials and Methods

### 2.1. Study Design

**Figure 1:**
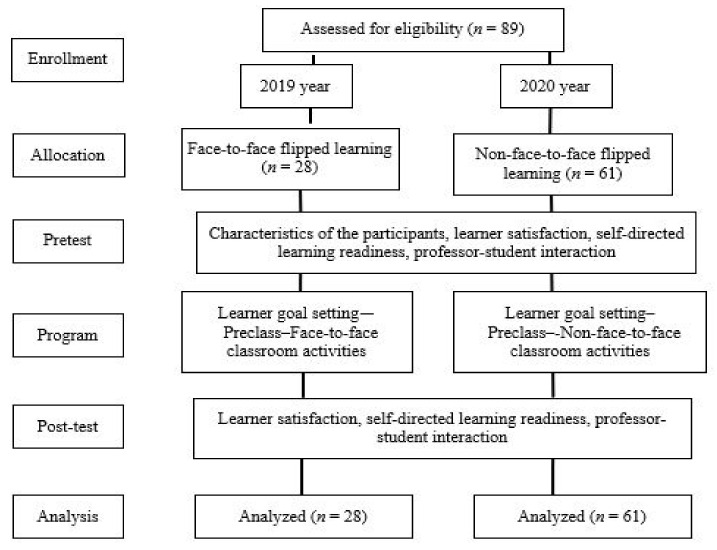
Flow diagram of the study (based on the CONSORT statement).

This study was conducted with undergraduate nursing students as a descriptive survey to identify the differences in self-directed learning readiness, professor–student interaction, and learner satisfaction after conducting face-to-face flipped learning and non-face-to-face flipped learning and identify the factors that affect the learning satisfaction of nursing students. The flow of the study process is shown in Figure 1.

### 2.2. Study Participants

The study participants included 89 s-year undergraduate students at a university who were taking course in a Nursing and English; those who understood the objectives of the study and provided voluntary consent were permitted to participate. Using the G*power version 3.1.2 [19] with a two-sided significance level (α) of 0.05, median effect size (*d*) of 0.15 for Cohen’s multiple regression, power (1-β) of 0.80 with four predictors, and a 10% dropout rate for the calculated number of participants, the target number of participants was 95. Participants were recruited at the orientation for a Nursing and English course; 28 students consented to participation in the study in 2019, and 61 students consented in 2020 for a total of 89 nursing students who consented to the participation in the study. The questionnaires were collected without any dropouts to be used in the final analysis. The final power was 0.97.

As the study was conducted as a questionnaire survey, there was no harm for the participants. However, as the participants were students enrolled in courses, best efforts were made to protect participants’ voluntary participation and personal information. At the orientation of the courses, the researcher first explained the objectives of the study, study method, data collection time points, and the students’ right to withdraw at any time without any academic penalty for refusing. Next, the students were informed that the data obtained from the questionnaire survey would not be used for purposes other than research.

### 2.3. Class Delivery Method

The study was conducted while ensuring that the key factors of flipped learning, such as student-directed approach, asynchronous content delivery, and the learning method combining information technology [8], were faithfully followed for face-to-face and non-face-to-face groups. The methods were identical with the exception of the in-class part in flipped learning; the specifics of the procedures are as follows.

#### 2.3.1. Pre-Class

##### Provision of Class Materials

In both face-to-face and non-face-to-face flipped learning, the instructor provided the materials to be learned during class through eCampus Blackboard. The eCampus Blackboard is a web-based learning system and electronic community center for students and faculty. The instructor provided videos of medical terminology to be learned for each week, with the native English speakers repeating each word twice along with the instruction materials on a Nursing and English for communication on the job as the foundation for learning required courses for the major as a way of providing various types of materials to enable a more efficient pre-class learning. This course is a sophomore subject regarding some basics of nursing—learning medical terminology for communication; thus, it has little direct relevance to learning direct nursing skills. Therefore, communication-related videos and terms that can be used in nursing practice were used as online content.

##### Preliminary Learning

In both face-to-face and non-face-to-face flipped learning, the instruction video was assigned as a required learning material among various types of pre-class learning materials provided. The learners were allowed to adjust and manage the quantity, pace, and hours of studying for themselves. The instruction materials and the terminology videos were provided to help the learners use their free time to learn according to their own circumstances and learning schedule. This pre-class learning method allowed for a student-directed approach and asynchronous content delivery.

##### Practice Exercises

In both groups, students were given practice exercises from the textbook to complete after the pre-class. The practice exercises in the textbook were given to ensure that the students completed the required learning [20] as online learning is performed by the students at home.

#### 2.3.2. In-Class

##### Quizzes

Quizzes were completed in class. For the face-to-face flipped learning group, the instructor administered weekly 10-min online quizzes consisting of 10 questions (five multiple-choice, five on accurate spelling of medical terminology) in eCampus Blackboard to check that the students completed the pre-learning. For the non-face-to-face group, students were allowed to go online at their convenience to take the quiz within 10 min before and 30 min after the start of the class. A timer was set to 10 min; at that point, the online quiz was automatically terminated, and the answers had to be submitted. Additionally, the questions shown to the students were only displayed on the screen once in random order, and the students were not allowed to go back to the previous screen so that the previously submitted answers could not be revised, and the answers could not be shared among students.

##### Case Building

Case building was done in the class; for the face-to-face flipped learning group, the instructor put six students in each group and gave 30 min to create a patient case using the medical terminology learned for that week on patients’ symptoms, diagnosis, treatment, and nursing, while going around the classroom to provide instructions for each group. Once the students prepared the PPT presentation and uploaded it on the discussion board in eCampus Blackboard, 10 groups each gave a three-minute presentation on various cases and received feedback. For the non-face-to-face flipped learning group, once the instructor assigned groups and group activities, the students were allowed to discuss them in groups without supervision; feedback was provided as comments from the instructor and students on the PPT presentation uploaded to the discussion board in eCampus Blackboard.

##### Q&A

Q&A was a class activity; for the face-to-face flipped learning group, the instructor provided immediate feedback on the various scenarios and situations presented by the students, issues discovered during discussions and applications through case analysis, answers to practice exercises, and questions on online quizzes. Instead of answering each question directly on the online bulletin board, the gist of each question was summarized and provided as an additional explanation during face-to-face class. For the non-face-to-face flipped learning group, the questions uploaded on the weekly Q&A in eCampus Blackboard were answered by the instructor or learners in a comment. Questions posted on the online bulletin board were each given an answer.

##### Discussion

In both face-to-face and non-face-to-face flipped learning, the students uploaded the materials they wanted to share from the contents related to the weekly learning that they learned in addition after the pre-class learning using the weekly Q&A session. In addition, continuous discussion and Q&A were held online in weekly Q&A sessions on the materials to be tested until the end of the quiz. Learners responded first to the questions uploaded by the students, and the instructor provided an additional response before class activities or before quizzes in case the response needed correction or additional explanation.

#### 2.3.3. After Class

Follow-up learning was commonly applied in both face-to-face and non-face-to-face flipped learning. Students who scored less than 70 points in the online quizzes completed additional weekly learning and were asked to submit homework on additionally learned material in at least one sheet of A4 paper.

### 2.4. Study Tools

#### 2.4.1. Learner Satisfaction

Learner satisfaction was measured based on questionnaires developed by Jung and Lim [21] and revised to fit the purpose of the present study. The questionnaires consisted of ten items, with the two subcategories of general learner satisfaction and academic learner satisfaction that were scored on a five-point Likert-type scale from “highly satisfied” (score of 5) to “never satisfied” (score of 1), with mean scores ranging from 1 to 5; a higher score indicated higher learner satisfaction. Cronbach’s for this questionnaire was 0.870 in Jung and Lim [21], and the reliability of overall questionnaire was 0.904, the general learner satisfaction 0.877, and the academic learner satisfaction 0.830, respectively, in the present study.

#### 2.4.2. Self-Directed Learning Readiness

Self-directed learning (SDL) readiness was measured using the 32-item Korean version of the Self-Directed Learning Readiness Scale revised by Kim et al. [22] and originally developed by Guglielmino [23]. SDL readiness consists of 32 questions in six sub-domains, including attachment to learning, self-confidence as a learner, openness to challenges, curiosity for learning, self-understanding, and acceptance of responsibility for learning. Scores were measured on a five-point Likert-type scale from “highly satisfied” (score of 5) to “never satisfied” (score of 1), with the mean score ranging from 1 to 5; a higher score indicated higher SDL readiness. In Kim et al.’s [22] study, the reliability of the scale in Cronbach’s α was 0.930; in the current study, the reliability of the six sub-domains were 0.756, 0.830, 0.761, 0.793, 0.745, and 0.704, respectively, and the reliability of the overall tool was 0.861.

#### 2.4.3. Professor–Student Interaction

For professor–student interaction scale, the Questionnaire on Teacher–Student Interaction developed by Fisher [24] was modified and supplemented by Hyun et al. [25] for use. The professor–student interaction scale consists of 18 questions in total, with two sub-factors: intimacy and reliability. Scores were measured on a five-point Likert-type scale from “highly satisfied” (score of 5) to “never satisfied” (score of 1), with the total scores ranging from 18 to 90 and a higher score indicating more satisfying professor–student interaction. In Hyun et al.’s [25] study, the reliability of the scale in Cronbach’s α was 0.920; in the current study, it was 0.942, and the reliability of the two sub-factors were 0.878 and 0.942, respectively.

### 2.5. Data Collection

Data collection was conducted in a time difference design from 4 March 2019 to 14 June 2019 and from 16 March 2020 to 24 June 2021 with second-year students taking Nursing and English courses. In preliminary data collection, the researcher explained the objectives and method of the study as well as the timing of questionnaires during the orientation of the course. The questionnaires were completed by accessing the URL for preliminary questionnaire on the e-campus notice. The start screen of the questionnaire URL provided the information sheet and the informed consent form on the objectives of the study, class method, rights of the participants, and personal information protection. Accessing the questions on the questionnaire on the following page was allowed only if the students read the information sheet prior to starting the questionnaire and provided voluntary consent to study participation. Follow-up data collection was conducted by uploading the URL for the follow-up questionnaire on the e-campus notice after the final examination and sending the same URL to the students taking the courses via e-mail. To complete data collection in a short period, the student president of each department put up a notice on SNS requesting all those who responded to the preliminary questionnaire to participate; it was conducted through voluntary connection.

### 2.6. Data Analysis

Data analysis was conducted using IBM SPSS Statistics 25.0 program (IBM, Armonk, NY, USA). The characteristics of nursing students, learner satisfaction, self-directed learning readiness, and professor–student interaction were analyzed using means, standard deviations, frequency, and percentages. The preliminary test of homogeneity for characteristics of nursing students, learner satisfaction, self-directed learning readiness, and professor–student interaction was tested using the chi-square test before implementing flipped learning. After applying flipped learning, the differences in the learner satisfaction, self-directed learning readiness, and professor–student interaction between the face-to-face and non-face-to-face groups were analyzed using the independent *t*-test. The difference obtained by subtracting the pre-values from the post-values to correct for the pre-values in the post-value comparison for self-directed learning readiness was tested; professor–student interactions were found to be non-homogeneous in the preliminary test. The difference in learner satisfaction according to characteristics of nursing students was analyzed using the independent *t*-test, whereas the correlations among learner satisfaction, self-directed learning readiness, and professor–student interaction were analyzed using Pearson’s coefficient correlation. The characteristics of nursing students, self-directed learning readiness, professor–student interaction, and the effects of face-to-face flipped learning and non-face-to-face flipped learning on learner satisfaction were analyzed using the stepwise multiple regression analysis. The significance level of each statistic was selected from *p* < 0.05.

## 3. Results

### 3.1. Participant Characteristics

Overall, 77 (86.5%) of the participants were less than 22 years old, and 72 (80.9%) were women. In the last semester, 49 participants (55.1%) had a mean GPA of 3.52. Prior to the flipped learning class, there was no difference in the participant characteristics between the face-to-face and non-face-to-face flipped learning groups (Table 1).

### 3.2. Differences between Face-to-Face and Non-Face-to-Face Flipped Learning Groups after Flipped Learning

Before the flipped learning class, self-directed learning readiness, and professor–student interaction were not homogeneous between the face-to-face and non-face-to-face flipped learning groups. To correct for this non-homogeneity in self-directed learning readiness and professor–student interaction in advance, the difference in post-values and pre-values was compared. As a result, there was no difference in self-directed learning readiness between the two groups after flipped learning (*t* = −1.15, *p* = 0.258). After flipped learning, the professor–student interaction was higher for the non-face-to-face flipped learning group than for the face-to-face flipped learning group, with statistical significance (*t* = −4.31, *p* < 0.001). After flipped learning, the learner satisfaction in nursing students was higher in the face-to-face flipped learning group than in the non-face-to-face flipped learning group with statistical significance (*t* = 5.28, *p* = 0.024; Table 2).

### 3.3. Difference in Learner Satisfaction According to Participant Characteristics

After flipped learning, the learner satisfaction in nursing students was higher for students with a mean SDL readiness score of 87.24 or higher compared to those with a score of less than 87.24 for total scores (*t* = −3.04, *p* = 0.003), general learner satisfaction (*t* = −2.84, *p* = 0.006), and academic learner satisfaction (*t* = −2.82, *p* = 0.006). There was no difference in learner satisfaction depending on other participant characteristics or variables (Table 3).

### 3.4. Correlations among Learner Satisfaction, SDL Readiness, and PSI after Flipped Learning Class

After flipped learning, the learner satisfaction in nursing students revealed that total scores, general learner satisfaction, and academic learner satisfaction were positively correlated with SDL readiness and PSI (Table 4).

### 3.5. Factors Affecting Learner Satisfaction after Flipped Learning Class

Table 5 shows the regression model for identifying the factors that affect learner satisfaction of nursing students after the flipped learning class. Gender, a categorical variable, was treated as a dummy variable, and age, last semester’s grades, SDL readiness, and PSI were entered as continuous variables and analyzed by a stepwise multiple regression method. When constructing the model, variables were selected based on a significance probability of 0.05, and variables were removed based on a significance probability of 0.10. In the learner satisfaction model, the tolerance limits between independent variables were all above 0.1, and the variance inflation index (VIF) also satisfied the criteria of less than 10, indicating that there is no problem of multicollinearity.

In the regression model for learner satisfaction of nursing students after flipped learning class, face-to-face flipped learning method (*t* = 2.39, *p* = 0.019), SDL readiness (*t* = 4.97, *p* < 0.001), and PSI (*t* = 2.44, *p* = 0.017) were significant influencing factors, and the explanatory power of the regression model constructed with these three variables was 36.7% (*F* = 18.00, *p* < 0.001). In the regression model for general learner satisfaction, a subdomain of learner satisfaction, SDL readiness (*t* = 4.76, *p* < 0.001), and PSI (*t* = 2.40, *p* = 0.019) were significant influencing factors, and the explanatory power of the regression model constructed with these two variables was 22.5% for general learner satisfaction (*F* = 16.90, *p* < 0.001). In the regression model for academic learner satisfaction, SDL readiness (*t* = 5.25, *p* < 0.001) and PSI (*t* = 2.47, *p* = 0.015) were significant influencing factors, and the explanatory power of the regression model constructed with these two variables was 33.3% for academic learner satisfaction (*F* = 22.97, *p* < 0.001).

## 4. Discussion

This study was conducted to examine the difference in self-directed learning readiness, professor–student interaction, and learning satisfaction for nursing students who had face-to-face classes with flipped learning and others who had non-face-to-face classes with flipped learning to identify the factors that affect learner satisfaction. First, self-directed learning readiness was improved for both the face-to-face and non-face-to-face flipped learning groups compared to before the class, but there was no significant intergroup difference. The lack of intergroup difference seems to be because the pre-class process was identical for both groups, as the students had to conduct self-directed learning in advance. Second, flipped learning allows for both student-directed approach and asynchronous content delivery through the learner’s preliminary learning [8], and these two factors were the common factors for both the face-to-face flipped learning and non-face-to-face flipped learning. Therefore, it seems that self-directed learning readiness did not show a significant difference between the two groups.

Results of this study revealed that professor–student interaction was significantly higher for non-face-to-face flipped learning than face-to-face flipped learning. In contrast, previous studies reported that it is difficult to ask questions of the instructor or obtain answers to questions during an online class [26] and that there is a lack of instructor–learner interaction online [27]. In general, it is believed that professor–student interaction is difficult in non-face-to-face classes due to not being in the same physical space. In learning, professor–student interaction is perceived as important by both the instructor and the learner [28], but the interaction can be perceived differently by each, and the learner’s perception of interactions is particularly important. In fact, a previous study [29] reported that the professor–student interaction as perceived by the learner encourages active participation of students and frequent interaction of communication, activities, and mutual interest. The method of interaction achieved in this study in relation is described below.

Both groups had the online bulletin board for mutual communication, but they were implemented differently to suit the class format. For the face-to-face flipped learning group, responses were provided during face-to-face class activities by pooling the questions together rather than answering each question. For the non-face-to-face flipped learning group, responses to questions were individually provided on the bulletin board. In the case of non-face-to-face flipped learning, there was a high degree of individual interactions, which could have been perceived as higher interaction by the learners, as reported by Kwon [30]. The reported results of that research regarding the interaction with online learners were that the quantitative increase in the interactive behavior through the Q&A bulletin board and posting contents directly related to the class were perceived as an increase in interactions. Owing to the nature of the class format, online interaction is dependent on the medium in a non-face-to-face class. The characteristics of online interaction include non-linearity, impracticality, many-to-many, recordability, multi-content-based, and information-rich communication [31]. 

Additionally, online interactions do not require immediate and linear interactions like face-to-face situations, and they allow sufficient time to organize thoughts for interaction [16], which could be perceived as more comfortable for some. From this perspective, it would be necessary to measure whether the learner feels that the mutual interaction is present rather than focusing on the methodology of face-to-face or non-face-to-face. Rather than assuming that there would be limitations in professor–student interaction for non-face-to-face learning, it would be necessary to maximize active professor–student interaction by optimizing the advantage of the communication using online media. Demonstrations of active professor–student interactions achieved in the non-face-to-face method suggest that such interactions can be promoted if the online system and the means for communication are open. 

In this study, learning satisfaction was higher for face-to-face than non-face-to-face flipped learning. When the factors affecting the learning satisfaction were examined, learning satisfaction was high for learning format, that is, for face-to-face flipped learning, when the self-directed learning readiness and the professor–student interaction were high. When examined individually, the details are as follows: 

First, class format, such as face-to-face format, had a positive effect on learning satisfaction. In flipped learning, online content learning occurred in the pre-class step, whereas the various learner-oriented activities occurred during the in-class step, and the role of the instructor became that of an advisor and facilitator providing feedback rather than that of a traditional instructor [8]. This instructor role was more efficiently achieved in the face-to-face class. Additionally, there may be factors, such as immersion, that could improve learning satisfaction for face-to-face compared with the non-face-to-face classes, as difficulties with concentration and immersion were reported in non-face-to-face learning [4]. When university students listen to online lectures for a long time, concentration decreases, and immersion in learning becomes difficult due to distractions in the surroundings, such as internet searches, games, YouTube videos, and webtoons that pop up as the computer is turned on [4]. 

Moreover, the difference in immersion to learning between face-to-face and non-face-to-face flipped learning could cause a difference in learning satisfaction. Previous studies reported that higher ability for immersion was associated with higher academic self-efficacy, which is the belief that one can perform well in learning [32]; immersion in learning leads to a better understanding of learning materials and, thereby, leads students to challenge themselves with higher-level tasks due to their confidence [33]. Furthermore, online lectures have the advantage of being repeated at the student’s convenience. In fact, it was shown that learners prefer video classes that they can watch repeatedly [34]. However, it could also suggest that the learners’ convenience does not increase learning satisfaction, per se. Additionally, non-face-to-face learning activities have the limitation of eliminating some kinds of nonverbal communication [35]. In text-based interactions, which comprise most online interactions, facial expressions, eye movement, and physical movements cannot be communicated; even if they could be delivered through the screen, they cannot be delivered in the same way as when in the same physical space [15]. These aspects result in the differences between the face-to-face and non-face-to-face learning environments, eventually leading to the difference in the level of satisfaction. 

The results of this study also show that students with high self-directed learning readiness had higher learning satisfaction. This finding supports the result of the previous studies [36] that reported the effects of self-directed learning readiness on learning satisfaction. Unlike traditional learning, online learning occurs while the instructor and the learner are in different times and spaces; thus, it is difficult for the instructor to manage the learning for the learners. Therefore, self-directed learning, which is the ability to plan and learn without the help of others in any type of circumstances and context, can be regarded as an essential parameter for improving performance in online classes [4].

In addition, learning satisfaction was higher when there was higher professor–student interaction. This finding is consistent with the reports [36] that learning satisfaction increases with increased professor–student interactions and the study that reported that communication with the professor and Q&A with the professor affected the online class satisfaction in terms of interactions during an online class [37]. As professor–student interactions are emphasized in flipped learning [7,8,18,19], it is necessary to promote such interactions. By maximizing the flexibility and the efficiency of flipped learning characterized by professor–student interactions and individualized learning, students’ academic achievement can be improved, and the flexibility and efficiency of learning can be increased, as prior online learning can be adjusted to suit the individual’s situation [8]. Considering these advantages, flipped learning can be applied flexibly according to circumstances, as it is believed that such learning can be transformed and applied according to the characteristics of the subject [38].

With the recent increase in the quantity of knowledge and information and in a situation that demands the ability to resolve complex issues, the importance of self-directed learning ability, which is the ability to efficiently use the various types of information, knowledge, and techniques obtained, is being emphasized. Based on the finding that self-directed learning readiness with increasing importance is associated with learning satisfaction, the flipped learning instruction method that could improve self-directed learning readiness needs to be applied flexibly depending on the time and situation. 

The results of this study could help in the design and implementation of interactions for effective online education in universities where the demand for online learning is increasing rapidly. Further, it may provide foundational knowledge for establishing a class strategy using face-to-face and online learning optionally or combining both. This study was applied with classes on theory-related subjects. However, the research can be sufficiently expanded to apply to clinical learning-related fields. Although there is a difference between theory and clinical practice, non-face-to-face practical subjects can also be conducted; thus, both factors that affect learning satisfaction and the importance of immersion and student interaction can be similarly applied to clinical field education.

## 5. Conclusions

This study was conducted to understand how flipped learning can be modified and to examine strategies for making it equally effective in face-to-face and non-face-to-face class activities. Various online instruction methods can be applied that utilize the advancements in technology to overcome barriers and better address crises, such as the COVID-19 pandemic. Results revealed that there was no difference in self-directed learning readiness between face-to-face flipped learning and non-face-to-face flipped learning; the professor–student interaction was higher for non-face-to-face learning, whereas learning satisfaction was higher for face-to-face learning. Furthermore, when the factors affecting learning satisfaction were examined, learning satisfaction was high for learning type, specifically face-to-face classes, when the self-directed learning readiness and the professor–student interaction were high. 

These results suggest that enhancing self-directed learning ability should be raised as an essential piece of the educational agenda and that professor–student interaction can actively occur in non-face-to-face environments. Therefore, further research is needed to develop methods to (1) include immersion in non-face-to-face education, as that is the most significant advantage of face-to-face classes; (2) include nonverbal communications; and (3) increase interactions among students in non-face-to-face classes. In addition, this study may provide the foundational knowledge for establishing class strategy while flexibly implementing flipped learning to suit the situations and the environment.

In this study, we found that it is necessary for both professors and learners to be flexible in coping with face-to-face and non-face-to-face teaching methods. Considering that convenience is not necessarily connected with learning satisfaction, we recommend creating an immersive learning environment regardless of the teaching method. Additionally, the findings support a recommendation for creating a learning environment wherein non-face-to-face activities are configured according to the characteristics of learners familiar with non-face-to-face learning and in which learners can actively participate.

## Figures and Tables

**Table 1:** Characteristics of the participants and homogeneity of the variables between the two participant groups (*n* = 89).

Characteristics		Total (*n* = 89)	Face-to-Face Flipped Learning (*n* = 28)	Non-Face-to-Face Flipped Learning (*n* = 61)	Χ^2^ or *t* (*p*)
		*n* (%)			
Age (year)	<22	77 (86.5)	22 (78.6)	55 (90.2)	2.21 (0.137)
	≥22	12 (13.5)	6 (21.4)	6 (9.8)	
	Mean ± SD	20.87 ± 1.67	20.96 ± 1.84	20.82 ± 1.61	−0.38 (0.707)
	Min. ~ Max.	19~28	19~27	19~28	
Gender	Male	17 (19.1)	3 (10.7)	14 (23.0)	1.86 (0.248) *
	Female	72 (80.9)	25 (89.3)	47 (77.0)	
Last semester grade	<3.52	40 (44.9)	11 (39.3)	29 (47.5)	0.53 (0.467)
	≥3.52	49 (55.1)	17 (60.7)	32 (52.5)	

* Fisher’s exact test.

**Table 2:** Comparison of variables in the two groups after flipped learning (*n* = 89).

Variables		Face-to-Face Flipped Learning (*n* = 28)	Non-Face-to-Face Flipped Learning (*n* = 61)	*t* (*p*)
		Mean ± SD		
SDL readiness	pretest	3.54 ± 0.32	3.27 ± 0.39	3.23 (0.002)
	post-test	3.64 ± 0.45	3.47 ± 0.43	
Difference (post–pre-test)		0.11 ± 0.41	0.20 ± 0.27	−1.15 (0.258)
PSI	pretest	4.25 ± 0.41	3.77 ± 0.54	4.19 (<0.001)
	post-test	3.97 ± 0.49	3.91 ± 0.50	
Difference (post–pre-test)		−0.03 ± 0.42	0.46 ± 0.53	−4.31 (<0.001)
Learner satisfaction	post-test	4.08 ± 0.55	3.70 ± 0.60	5.28 (0.024)

Abbreviations: SDL, self-directed learning, PSI, professor–student interaction.

**Table 3:** Differences in learner satisfaction according to participant characteristics (*n* = 89).

Characteristics		Learner Satisfaction (Total)		General Learner Satisfaction		Academic Learner Satisfaction	
		Mean ± SD	*t* (*p*)	Mean ± SD	*t* (*p*)	Mean ± SD	*t* (*p*)
Age	<22	3.80 ± 0.57	−0.75 (0.453)	3.70 ± 0.66	−1.21 (0.230)	3.87 ± 0.59	−0.33 (0.744)
	≥22	3.94 ± 0.80		3.96 ± 0.90		3.93 ± 0.76	
Gender	Male	3.91± 0.71	0.70 (0.485)	3.88 ± 0.74	−0.98 (0.328)	3.93 ± 0.75	−0.41 (0.683)
	Female	3.80 ± 0.58		3.70 ± 0.69		3.86 ± 0.58	
Last semester grade	<3.52	3.77 ± 0.60	−0.66 (0.514)	3.70 ± 0.72	−0.40 (0.687)	3.82 ± 0.59	−0.77 (0.442)
	≥3.52	3.86 ± 0.61		3.76 ± 0.68		3.92 ± 0.63	
SDL readiness	<3.36	3.64 ± 0.57	−3.04 (0.003)	3.54 ± 0.67	−2.84 (0.006)	3.71 ± 0.56	−2.82 (0.006)
	≥3.36	4.01 ± 0.59		3.94 ± 0.67		4.06 ± 0.62	
PSI	<3.92	3.71 ± 0.56	−1.72 (0.088)	3.64 ± 0.68	−1.22 (0.226)	3.76 ± 0.57	−1.91 (0.059)
	≥3.92	3.93 ± 0.63		3.82 ± 0.71		4.00 ± 0.64	

Abbreviations: SDL, self-directed learning, PSI, professor–student interaction.

**Table 4:** Correlations among the variables (*n* = 89).

Variables	Learner Satisfaction	General Learner Satisfaction	Academic Learner Satisfaction
		r (*p*)	
Learner satisfaction	1		
SDL readiness	0.56 (<0.001)	0.48 (<0.001)	0.55 (<0.001)
PSI	0.36 (0.001)	0.28 (0.008)	0.37 (<0.001)

Abbreviation: SDL, self-directed learning, PSI, professor–student interaction.

**Table 5:** Factors influencing learner satisfaction (*n* = 89).

Variables	Learner Satisfaction Total			General Learner Satisfaction			Academic Learner Satisfaction		
	B	SE	*t* (*p*)	B	SE	*t* (*p*)	B	SE	*t* (*p*)
Intercept	4.37	5.24	0.83 (0.406)	4.63	2.07	2.24 (0.028)	2.13	3.27	0.65 (0.516)
Face-to-face flipped learning *	2.67	1.12	2.39 (0.019)						
SDL readiness	0.24	0.05	4.97 (<0.001)	0.11	0.02	4.76 (<0.001)	0.15	0.03	5.25 (<0.001)
PSI	0.15	0.06	2.44 (0.017)	1.33	0.55	2.40 (0.019)	0.09	0.04	2.47 (0.015)
Adj. R^2^	0.367			0.225			0.333		
F (*p*)	18.00 (<0.001)			16.90 (<0.001)			22.97 (<0.001)		
Tolerance	0.87–0.96			0.97			0.91		
VIF	1.04~1.15			1.04			1.10		
Durbin–Watson	2.34			1.87			2.42		

* Reference: non-face-to-face flipped learning. Abbreviations: SDL, self-directed learning; PSI, professor–student interaction; VIF, variance inflating factor.
